# Research collaboration with older people as a matter of scientific quality and ethics: a focus group study with researchers in ageing and health

**DOI:** 10.1186/s40900-024-00540-y

**Published:** 2024-01-10

**Authors:** Synneve Dahlin-Ivanoff, Isak Berge, Emmelie Barenfeld, Maria Haak, Qarin Lood

**Affiliations:** 1 Institute of Neuroscience and Physiology, Department of Psychiatry and Neurochemistry, Sahlgrenska Academy, University of Gothenburg, Gothenburg, Sweden; 2 Centre for Ageing and Health – AgeCap, University of Gothenburg, Gothenburg, Sweden; 3 Centre for Person-Centred Care (GPCC), University of Gothenburg, Gothenburg, Sweden; 4 Institute of Neuroscience and Physiology, Department of Health and Rehabilitation, Sahlgrenska Academy, University of Gothenburg, Gothenburg, Sweden; 5 Research Platform for Collaboration for Health, Faculty of Health Science, Kristianstad University, Kristianstad, Sweden; 6 School of Nursing and Midwifery, Faculty of Health Sciences, La Trobe University, Melbourne, Australia

## Abstract

**Background:**

Society is placing increasing demands on collaboration with actors outside the academia to be involved in the research process, and the responsibility for turning this into reality lies with the researchers. As research collaboration is a way to increase the societal relevance of research and since older people have the right to be actively involved in research that concerns them, this study is addressed to researchers who work with and for older people. The purpose of this article is to explore researchers’ experiences of research collaboration with the heterogeneous group of older people, from healthy to frail.

**Methods:**

The focus group method was applied based on a qualitative approach that is based on a social constructivist research tradition. It differs from other qualitative methods, such as interviews, in that it encourages interaction between research participants and contributes to shedding light on a collective understanding of the world. A total of 14 researchers participated in four focus groups (three to five participants/group).

**Results:**

The results provided support for the overall theme: “Good scientific quality and ethics are balanced against the needs and abilities of older people”. This means a balance between the researcher and the older people collaborating with them to receive the best possible scientific quality. This is highlighted in the core category “Positioning for research collaboration” with the subcategories “Involvement or not”, “Traditional or innovative thinking” and “Selectivity or representativeness”, and the core category “Research collaboration – an ethical issue of power” with the subcategories “Research collaboration a risk for freedom of research”, “Research collaboration a risk of abuse of power” and “Discriminatory academic power structures create ethical issues”.

**Conclusions:**

Addressing the balancing act of collaborating with older people in research, the findings contribute with an understanding of the importance of researchers’ awareness of social and academic structures to minimise the risk of epistemic injustices in research on ageing and health. We want to highlight the researchers’ voice and clarify the role that researchers have in terms of the opportunities for older people to become part of the collective understanding of ageing and health and make their voices heard.

## Abstract (plain language summary)

Society is increasingly expecting researchers to involve people who are not researchers in their research. To understand how such collaboration could become a reality, this study aimed to explore researchers’ experiences of collaborating with older people in research on ageing and health. A total of four focus groups consisting of 14 researchers from two universities were conducted to discuss experiences, approaches, opportunities and obstacles for research collaboration with older people. The results revealed an overarching theme that describes research collaboration as a balancing act with scientific quality and ethics on one side, and the needs and abilities of older people on the other side. This means that researchers need to strike a balance between achieving the highest scientific quality and considering the needs and abilities of older people they are collaborating with. To understand how unethical, it is to not involve older people in research, the concept epistemic injustice has been used. It refers to the systematic exclusion of certain groups from knowledge production and dissemination which can lead to the exploitation of vulnerable populations and the perpetuation of harmful stereotypes. In addressing the challenges of collaborating with older people in research, this study emphasises the importance of researchers being aware of both social and academic structures that might affect whose voices are heard in research. This awareness could help researchers clarify their role in giving older people the opportunity to be part of the collective understanding of ageing and health.

## Background

Research collaboration with actors outside the academic world is encouraged at policy level both by the Swedish government [1] and by the European Commission [2]. This has led to the development of “Open science”, which stands for accessible and transparent shared knowledge. Open science means, among other things, actively involving non-scientists in the research process, which can be beneficial for several actors such as researchers, decision-makers, and citizens [3]. This has culminated in the question of how research collaboration with actors outside the world of the academia should be performed. Research collaboration has become an increasingly common requirement that must be dealt with in applications for research funding [4]. Universities today are also under increased demands to contribute to the surrounding society in addition to education and research – the so-called third mission of universities [5]. Those who then are given the responsibility for turning these policy decisions into reality are the researchers.

In an analysis of the European Commission’s policies in the years 1998–2019, Macq et al. [6] describe how initiatives have been taken to open up the production of knowledge and innovation. During this period, there have been two different discourses on public participation that sometimes contrasted with each other. The first discourse is about increasing the participation of citizens in decision-making to increase the legitimacy of decisions made by society’s scientific and political institutions. The second discourse is about the participation of citizens in creating innovations of services and products to increase the competitiveness of the member states in the global economic market. In the midst of these ongoing discourses has been the academic world and, ultimately, the research community [6]. This shows that the view of the production of knowledge is changing, and it affects the way researchers work and how their work is viewed.

Collaborating in research with actors outside the academic world is part of several different research traditions with different names, such as user-driven research, community-based participatory research, co-design, co-production of knowledge, patient and public involvement, patient-driven research, transdisciplinary research, and collaborative research [7–9]. Common to these different concepts or research traditions is that participants are involved in research as more than just data sources [10, 11]. Research collaboration can take place in all parts of the research process, in everything from planning the study, collecting data, analysing the data, and disseminating the results [11]. It is a matter of mutual respect and the sharing of influence and power by recognising the knowledge of all actors, both the researcher and those with whom they are collaborating. In this way, new areas of knowledge can be created for a new kind of knowledge production [12]. Seen from the perspective of the target group of frail older people, our previous research has shown that they both want to and can be involved in research, but that living with frailty involves morbidity and disability, which affects the conditions for how and when it is possible to be involved in research. This means that it can be difficult for researchers to determine for themselves in advance when and how frail older people can collaborate in their research. This decision needs to be made in dialogue between researchers and frail older people [13]. Researchers in healthcare research must also adhere to the ethical principles of beneficence, justice, and autonomy to ensure that research is fair and does not harm participants. It is also important that the research is of high quality and that the results are reliable and useful for improving care. Research ethics is about protecting the dignity, rights, and well-being of research participants by following ethical principles. It is important to follow these principles to ensure that the research is fair and does not harm the participants. In healthcare research, it is important that the research is ethically correct and that the research participants are protected [14].

Research collaboration with older people in healthcare science research is important to ensure external validity. It means applying the results of the research in a wider context that reflects reality [15]. Among the most common motives for involving actors outside the academia in research are to ensure that the research is relevant to the target group, to strengthen the validity and credibility of the results, and to increase the possibilities for implementation [16]. Research collaboration has also already more than ten years ago described as being able to increase the relevance of research to society and reduce the distance between theory and practice [11, 12]. When research collaboration instead comes as a requirement when applying for research funding, there is a risk that it becomes a box to be ticked rather than something that has a real influence on the research process [17, 18].

Within research on ageing and health, research collaboration is an important issue because both age and frailty have been shown to have a negative impact on research with and for older people. At the same time, there is a large heterogeneity within the older population, with large individual differences, ranging from healthy to severely frail [19, 20]. Walker argued already in 2007 that older people have the right to be actively involved in research on ageing [21]. In contrast, a recent survey [16] shows that there seems to be no consensus among researchers in ageing and health in Sweden on whether or how actors outside should be involved in research [16]. In a Swedish report from 2019 [22], researchers from several different research fields answered that they are partly positive about transparency towards the outside world in the various parts of the research process, but sceptical about the outside world influencing the research. If the outside world is to have insight into and/or influence research, researchers prefer that it happens at the beginning of the research process (prioritisation of areas and funding) and at the end (use of results) [22]. This has also been highlighted internationally, including by Boylan et al. [18] who describe that a higher degree of involvement in research increases the researcher’s emotional investment, for both good and bad. It is felt to contribute to increased motivation and a feeling of making a difference for other people, but also to a feeling of increased stress and heightened responsibility. The researchers also felt threatened that outside actors would gain influence over their research and how this could threaten their independence [18]. It is important to avoid instrumentalizing in research which refers to the practice of treating research participants as mere instruments or tools to achieve a specific goal, rather than as human beings with their own agency and dignity. This approach can have serious consequences for both researchers and the quality of research. This can lead to a lack of trust between researchers and participants, which can ultimately undermine the quality of the research. [23].

Society is increasingly demanding that actors outside academia be involved in the research process. Open science encourages collaboration between all relevant and interested open science stakeholders across the world, including public and private science, technology and innovation institutions, relevant private sector and industry, United Nations agencies, and all other relevant open science actors. There is also a growing demand for the academia to engage in participatory research, and the responsibility for turning this into reality lies with the researchers. Nevertheless, the academia faces several challenges such as lack of funding, infrastructure, academic culture, and lack of incentives. It is problematic to exclude frail older people a priori, and there is potential for new perspectives and knowledge to be created in collaboration. However, in what way and how is difficult to know before they are involved in the research process where they, in collaboration with researchers, get the opportunity to feel out what they want and can do at the moment [13]. Researchers’ experiences of research collaboration with a heterogeneous group of older people, from healthy to frail, is very limited. Research [24] have found that participatory research can help researchers better understand people with dementia and broaden their theoretical knowledge and perspective. By collaborating with older people, researchers could increase the societal relevance of their research and improve its external validity and generalizability. This study aims to address the right of older people to be actively involved in research that concerns them. The purpose of the article is to explore researchers’ experiences of research collaboration with a heterogeneous group of older people, from healthy to frail.

## Design and methods

### Design

In this study the focus group method was applied to allow people to meet and discuss different aspects of a topic or theme in a focused way, led by a group leader [25, 26]. The focus group method generates knowledge based on shared experiences and focuses on the variation in the collective understanding that emerges from the discussion [27, 28]. The focus group method stem from a qualitative approach that is based on a social constructivist research tradition [27, 29]. It differs from other qualitative methods, such as interviews and observations, in that it encourages interaction between research participants [30] and contributes to shedding light on a collective understanding of the world [27].

## Participants

A total of 14 researchers participated in four focus groups (three to five participants/group). When creating dynamic focus groups, both homogeneity and heterogeneity were considered when choosing participants. Homogeneity is about sharing similar experiences to create discussion. In this study, this meant that participants shared the experience of conducting focus groups covering healthy older persons to frail older persons. Heterogeneity is needed to cover diversity within the chosen target group to allow for reflection upon each other’s experiences and was considered in all focus groups through a form of purposeful sampling. Thus, the researchers represented different disciplines, such as the humanities, social science, medicine, nursing, occupational therapy, physiotherapy, and healthcare science, and came from two different universities in southern Sweden. At each university one focus group with senior researchers (professors and associate professors) and one with junior researchers (PhD students and researchers who had not yet become associate professors) were conducted.

## Procedure

The focus group discussions were conducted in university conference rooms. Each group session lasted no more than 1.5 h and was led by one moderator, experienced in conducting focus groups (QL associate professor, last author, all groups) and two different co-moderators (SDI professor, first author, focus group 1, IB PhD student, focus group 2–4). The moderator led the discussions while the co-moderator observed, took field notes, and asked follow-up questions when needed. The discussions were based on four key questions developed for the aim of this study: 1) experiences of and 2) approaches to research participation as well as 3) opportunities and 4) obstacles for research participation about and with older people. The sessions began with the moderator informing the participants about the study aim and the structure of the focus group. The participants introduced themselves and told the other group members a little bit about themselves. The moderator then introduced the discussion topic, and the participants were encouraged to discuss the topic openly. The moderator’s task was to pose questions to deepen the discussion and to ensure that all participants were given a chance to speak, identifying common elements in the discussions and posing general questions followed by more specific ones. All sessions were audio-recorded and transcribed verbatim for analysis.

## Data analysis

The analysis was based on a method developed by Kreuger and Casey [26]. To keep the raw data in view long enough to understand the meaning of the material, the analysis was conducted in Swedish as far as possible. In the first step, the researcher uses raw data to try to get an overall idea of the entire content of the material. To become familiar with and gain an understanding of the content of the data material in its context, the first step in the analysis was to listen to the audio recordings several times. The transcript of each focus group session was then read carefully and independently to get an overall sense of the data. After listening to the raw material several times and reading through the transcribed material, along with the notes taken, preliminary themes that were consistent throughout the data were identified. Next, sections relevant to the research topic were identified and sorted into different themes, guided by the aim of the study to comprehend the contextual meaning of the material. Categories were then defined from a review of the raw data, and descriptive statements that synthesised, abstracted, and conceptualised the data were constructed. The next step was to systematise the raw data under the identified themes into categories, that is, to place the actual discussions in an appropriate category. The parts, i.e. themes and categories, were continuously related to the whole, which provides a continuous revision of themes and categories. The purpose of this phase is to create themes and categories that correspond to meaning.

The last step was to summarise the categorised raw data, combined with an interpretative step aiming to provide an understanding of the participants’ discussions. The analysis process is described as a continuum where the raw data, consisting of the exact and raw discussions, were categorised and concentrated into descriptive summaries. The descriptive summaries then formed the basis for the interpretation of the data. The data analysis process was iterative: each step was initially conducted by the first author separately and discussed with the second author in a back-and-forth process to not lose the connection with the raw data. The last author had the role of triangulating the interpretative step to enhance the credibility of the findings. Throughout the analysis, the shared experience and the collective statements that were shaped, and reshaped, formed the basis for the understanding of what was being studied and the final interpretation was agreed upon by all authors.

## Findings

### Good scientific quality and ethics are balanced against the needs and abilities of older people

The results provided support for the overall theme: “Good scientific quality and ethics are balanced against the needs and abilities of older people”. Research collaboration with older people means highlighting the research problems most important to them and giving them the opportunity, based on their abilities and needs, to be part of the decision-making process in the issues that concern them. This means striking a balance with a focus on when, how, and which older people can, want to, and should be included in the best way, as well as considering the research ethical issues that arise in research collaboration between the researcher and the older persons to receive the best possible scientific quality. This is highlighted in the following two core categories: “Positioning for research collaboration” and “*Research collaboration – an ethical issue of power*”, both with subcategories (see Table 1).

**Table 1:** Researchers’ experiences of research collaboration with older persons

Good scientific quality and ethics are balanced against the needs and abilities of older people					
Positioning for research collaboration			Research collaboration – an ethical issue of power		
Involvement or not	Traditional or innovative thinking	Selectivity or representativeness	Research collaboration at risk for freedom of research	Research collaboration a risk of abuse of power	Academic discriminatory power structures create ethical issues

### Positioning for research collaboration

The researchers in our study regard research collaboration as a prerequisite for gaining increased knowledge and understanding of the phenomenon that is to be studied. They believe that it is important that the researchers position themselves around when, how, and which older persons should be involved and to what extent. The degree of research collaboration among different researchers is related to what research is being conducted and where, and whether it is healthcare research or not. In the positioning process as a researcher, they found that one must make the best possible decisions regarding research collaboration with older persons to maintain as high a scientific quality as possible where each person’s abilities and needs are at the centre. When, how, and which older people should be involved in research collaboration is considered an important balancing act for researchers to achieve as good research collaboration as possible without compromising the scientific quality. The balancing act was highlighted through the following sub-themes: “Involvement or not”, “Traditional or innovative thinking”, and “Selectivity or representativeness”.

#### Involvement or not

The researcher must carefully consider which studies and phases of a research project are appropriate for collaboration, and with whom. It is important to strike a balance between studies that are beneficial for older people to be involved in and those that are not. Older people, like other groups, can be difficult to involve in research, and researchers ought to avoid preconceived notions that they cannot or do not want to be involved. It requires active awareness on the part of the researcher to avoid negative stereotypes. Researchers are responsible for ensuring that older people are acting according to their own free will, rather than living up to the demands of the researcher and the environment. They should also make it clear that older persons have something important to contribute to the production of knowledge. This is especially important for socioeconomically vulnerable and frail older people, who may be more difficult for researchers to involve in research.

#### Traditional or innovative

Good research collaboration with older people, according to our study participants, requires the researcher to strike a balance between doing things the way they have always been done and thinking innovatively. In a research project, it was described as important to identify older persons who can tell and express how they feel. The risk with this is that more hard-to-reach persons might be missed. Therefore, researchers should think innovatively, for example by being flexible and adaptable in terms of location, content, and how data collection is carried out. Choosing a research method is difficult in research with older people and this is a group that may need more time for reflection. The researcher needs to reflect on how the research collaboration with the older person needs to be carried out innovatively to be able to meet them based on their abilities and needs, even if it takes extra time and costs money. This is time-consuming work where sensitivity and the ability to listen are in the foreground so that the older person and the researcher can meet, and so that scientific and societal benefit meet as a basis for as high a scientific quality as possible.

#### Selectivity or representativeness

Our study participants pointed out that the people who collaborate in research do not always represent the older people the researcher wants to participate. The researchers often ask themselves, “Have we brought the right people with us?” The balancing act is about whether the researchers have succeeded in collaborating with a representative older person or not. Most often, it can be healthier older people from pensioner organisations, interest organisations, reference groups or, for example, people with higher education and high socioeconomic status who are asked about research collaboration, and that the older people who the researcher really want to involve do not want to participate or are not asked. It is not uncommon that people who cannot speak or read Swedish, or have poor hearing, poor vision, or a cognitive impairment, are not asked about research collaboration. This means a lack of representation in research that increases and reinforces the selectivity of the population the researcher wants to collaborate with. There is a great risk that it will be a selected group, probably not fully representative, and that the result to be applied for that group is not based on their vote.

*Quotation focus group 3* (senior researchers in medicine and health and care sciences).

RESPONDENT 2: I find it interesting to, to highlight when it is not appropriate. Because that's rarely what we talk about when it's not appropriate.

RESPONDENT 1: Mm.

RESPONDENT 1: yes, then I would … then there must be such here, eh, such representative groups as PRO (Swedish National Pensioners' Organisation) and SPF( The Swedish Association for Senior Citizens), for example….

RESPONDENT 3: that's often where we end up.

RESPONDENT 1: but it will not really be these we are going to investigate, but it will be one, a distance from them.

RESPONDENT 3: after all, it will be those who are verbal, who are used to speaking out, who are not afraid to converse in a group. And they do not represent the whole group. And often not the ones we are really interested in.

RESPONDENT 1: but maybe it will still be … maybe next best, then. Because that, it might be slightly better than without the older (persons) at all. They still have contacts. They have a network with other older people, and may know someone who has care and is related,

RESPONDENT 3: when we have interviewed older people about existential loneliness, and then we have interviewed someone who they themselves may indicate is close to them. And there we see that the experiences differ markedly. So that, yes, it is problematic.

RESPONDENT 2: yes, it is well where the relatives believe that a loved one is more lonely. Isn't that, right?

RESPONDENT 3: No, it is not. But, but the relatives, they think that their older, yes, mum, dad or so, need more activities. While the older ones themselves, they say it's not about the number of activities, it's about something that makes sense. They say their day consists of waiting. You wait for something all the time, and it's not like the life they really want to live, while those close to them see something else. Because it is from the inside and from the outside-perspective that is … so, we like to think that:”I know another person well." But then, that's often not the case.

### Research collaboration – an ethical question of power

The researchers experienced it as unethical not to involve older people affected by the research to participate in the production of knowledge that emerges in various types of research collaboration. The reason for collaborating in research should not be that it is modern, or that the research financiers require it, or that the researcher wants to be able to show how good they have been. Instead, it is about collaborating in research in different ways and striving for the research to be relevant for both parties. The research should respond to the needs of both researchers and older people, that is, fulfil the interests of the older people concerned and the need for research to respond to good scientific practice. The older people who the researchers collaborate with must feel that what they are doing is meaningful and that they can be part of a context that is important for the future. The research ethical issues that arise in research collaboration with older people are affected by different power structures that the researcher must deal with. This is highlighted in the following categories: “Research collaboration a risk for freedom of research”, “Research collaboration a risk of abuse of power”, and “Academic discriminatory power structures create ethical issues”.

## Research collaboration a risk for freedom of research

The researchers experienced that research collaboration with older people could pose a risk to the freedom of research. They suggest that this is due to the shift of power from researchers to the older person that the research collaboration creates. This shift of power could lead to professional scientific knowledge becoming secondary and the user’s voice being heard instead. When users have to step in and control how researchers interpret data, there can be a clash with good scientific practice which is seen as a potential risk for research collaboration.

## Research collaboration a risk of abuse of power

There is an ethical risk that those who say yes to the research collaboration do not really understand what they have said yes to. Researchers have a responsibility to make sure that those who choose or accept to collaborate in research really want to do so. This entails a risk that the researcher responsible for the study forces a “yes” because research collaboration in research is important. Researchers have an upper hand because they are the ones who start the research process, both when doing research together with older people and when researching them. Researchers should be aware of which processes can be triggered and pay attention to both verbal and non-verbal signals and use dialogue and responsiveness as tools so power relations can be equalised.

## Academic discriminatory power structures create ethical issues

Academia has discriminatory power structures, a form of discrimination which a researcher can feel unethical to be part of. These power structures continue without the possibility of questioning simply because they are established structures, and this is described as the way it always has been. One example of a discriminatory ethical issue is the researcher’s role in the information letters given to all people for ethical approval. The researchers in our study believe that the requirements have become so extensive that it may be unethical to put them in the hands of older people. The ethical approval risks creating structures that make it difficult, or even impossible, to support research collaboration with older people. As a researcher, it is important, as far as possible, to try to understand the obstacles to a fair representation. It is the researcher’s responsibility to shine a spotlight on the type of knowledge researchers strive for to capture the voice of the whole group of older people.

*Quotation focus group 2* (junior researchers’ media, psychology, medicine).

RESPONDENT 1: Yes, but also that there are so many, I sometimes think, very established structures within research, a way of working that you just keep on going, that just, um, what do you say, like, you may not question all the steps in the process, but “That’s how it’s always been done.” That it becomes some kind of convenience selection and, in method and process, partly of course for resource reasons, but perhaps also, well, because there is a, a pressure, that you should generate as quickly as possible. And then all those things become a problem, unfortunately, and as a researcher, yes, it looks very different across different disciplines, but often you are not alone, but you collaborate with others and there are many people who think and ponder. And as a junior researcher, you are often in a dependent position. And maybe you don’t always dare to question, come up with new proposals, because you trust or don’t dare to.

RESPONDENT 3: Say.

RESPONDENT 1: Say something else, like.

RESPONDENT 2: No, but absolutely, like.

RESPONDENT 1: Um, that makes you, you keep going.

RESPONDENT 2: And also the structure within academia that you still feel, like here that when you have sat like this: “Mm, now I’m going to, now I’m going to dare. Now I’m going to say.” So just, you get a little like this: we don’t have time for that", or: "Sometimes it can be silenced in a very terrible way like I said now. there is a small tendency to: “Yes, yes, we’ll take it another time.” Or like this: “It, it doesn’t work right now anyway”, or: “When you have the opportunity to apply for a little more money, then you can do that.”

## Discussion

The researchers in our study highlight research ethical issues that arise in the research process when balancing scientific quality against the needs and abilities of older people. Older people are a heterogeneous group [19], ranging from healthy to frail persons with morbidities and disabilities. An important task for researchers is therefore to give a voice to the potentially most vulnerable and frail older people in our society through the co-creation of legitimate research-based knowledge. When groups are excluded from research in which they have legitimate reasons to be involved, what Fricker [31] calls epistemic injustice occurs. It is about who are considered credible sources when new knowledge is to be formed. It then becomes important to develop equal opportunities for all older people to enjoy their human rights to make their voices heard through collaboration in research that supports dignified ageing among older people [32].

Older people can be a hard-to-reach group that is often excluded from being involved in research based on the general assumption that frailty, cognitive impairment, and morbidity have negative consequences for a person’s ability to contribute to “the scientific process” [33]. It has also been postulated that co-creation in research is not beneficial for older people with cognitive impairments who are often portrayed as lacking thoughts and desires worthy of being taken seriously [34]. The researchers in our study point out that every innovative effort needs to be made to strengthen opportunities for the people involved to participate in the research process. Research shows [35, 36] that a person-centred approach is required, which means that collaboration in research must be based on respectful dialogues that facilitate each person’s participation and self-determination, with consideration of personal integrity. In a recently published study [37] it is highlighted that researchers ought to reflect on their role and position in academia, question existing frameworks and whose knowledge is ruled out, and how and at what stage of the research process this occurs. It also means that people need to be treated as equals who have valuable knowledge. Mutual trust and security are needed because sharing and reflecting can cause discomfort and uncertainty. In a dialogue of this kind, the person concerned must be given the opportunity to express what is important to them. Researchers must open up the possibility of mutual reflection and the expression of the perceptions, feelings, and experiences that are needed to be able to discuss the value of dialogic reflections to share our stories about ethical issues [36, 37].

In addition, the researchers highlight that it can be healthier older people from pensioner organisations, interest organisations, reference groups or, for example, people with higher education and high socioeconomic status who are asked about research collaboration, and that the frail older people who you really want to involve are left out or excluded to take part in research, at all. Since it is the older persons, themselves are the ones who are the most legitimate decision makers on questions impacting their body and health, their insights and priorities should be of utmost relevance for society. The researchers made it clear that there is a great risk that it will be a selected group that probably is not fully representative, and that the results then are not based on the vote of the group on which they are to be applied. This is supported by a consensus report [38]. Among other things, the report highlights that the lack of representation endangers the generalisability of clinical research results and undermines trust in clinical research. They highlight the importance of efforts to create more representative and inclusive research environments to increase trust in science. Lack of representation exacerbates health disparities in the populations currently underrepresented and excluded from clinical trials and clinical research. The failure to achieve equity leaves health disparities unaddressed and further reinforces inequities [38]. Lack of representativeness is a challenge for almost all data collection methods. Whenever people do not have the chance or are not willing to be involved in research, or when some are overrepresented, the results can be skewed and biased [39], which the researchers in our study were well aware of in research both with and for older people.

The results show that the research ethical issues that arise in research collaboration with older people are affected by the different power structures that researchers have to deal with. Ethical issues are nothing new in research but a constant ongoing process that involves protection of the rights, safety, and wellbeing of the research participants [40]. One of the basic principles in the Declaration of Helsinki is that the care of the person must always come before the interests of science and society [40]. That research should be independent has long been a fundamental principle in Sweden [41]. Our results describe that the researchers feel that collaboration to increase older people’s visibility and their opportunity to influence could pose a risk to the freedom of research and thus a shift of power from researchers to the older person. The study shows that there is resistance among the researchers to the older persons collaborating in research having influence over the scientific analysis and the final interpretation of the results. This is confirmed by Boylan et al. [18] who highlight that researchers may feel threatened by non-researchers having influence over their work. This is also brought forward in a Swedish report [22] which shows that researchers in various fields can imagine some transparency and influence on their research from the public at the beginning and at the end of the research process, while these figures are markedly lower for the phases that concern the actual production of the research. However, Groot et al.[37] highlight that academic researchers and professionals do not have a monopoly on knowledge and ground this in Fricker’s [31] view of justice, where being heard and believed is seen a basic human right. This should be seen from the perspective of academic freedom, which is seen as a prerequisite for the development of society. New knowledge and new discoveries come about when researchers can follow their own ideas, thoughts, and goals [1]. In research on ageing and health open science makes high demands on researchers to navigate ethically to fulfil the values by respecting academic freedom and human rights and at the same time support high-quality research.

The study shows that researchers describe they have more power because they are the ones who start the research process when doing research together with older people. Even if the researcher has the best intentions and no hidden agenda, there is a risk of coercion – that vulnerable groups are forced into research collaboration they do not really want to be part of, or cannot bear, in the name of good research. Ensuring that no one is forced into involvement in research applies to all research, but perhaps especially research with and for frail older people. An example of this is the use of gatekeepers, such as healthcare staff in the recruitment procedure. Although establishing trust and building rapport are crucial for a successful recruitment process, the use of gatekeepers could implicate forced involvement if researchers are not careful with the information provided to staff. Haak et al.’s research [42] shows that frail older people experience research as something that someone else does and that they perceive the profession of researchers as standing above ordinary people. The same study showed that research was seen as difficult to access and understand for the general public [42]. The importance of creating a trusting, tolerant, and relaxed environment cannot be stressed enough if you want to get older people to share their experiences and knowledge. Being with others in a non-judgmental and permissive environment can empower older people to express their perspectives and thus distribute power between researchers and older people [43].

The researchers in our study describe how the structure of academia creates discrimination of vulnerable groups and the frustration and powerlessness they feel in the face of maintaining discrimination without being able to do anything about it. The frustration the researchers describe is reminiscent of the stress of conscience that health care workers describe experiencing in moral situations when they are prevented from acting or addressing those situations [44]. That research collaboration can mean stress for researchers is also described by Boylan et al. [18] as their study showed that researchers experience that emotions are mixed into the work to a greater degree in research collaboration. As the American philosopher Elizabeth Anderson [45] has pointed out in her influential article “The Democratic University: The Role of Justice in the Production of Knowledge”, universities are inevitably political institutions because exploring and investigating social phenomena involves a social endeavour and “what one believes crucially depends on whom one believes” [45]. This can also be interpreted based on Fricker’s [31] description of epistemic injustice, which ranges from signs of ruling technology in research situations to injustices that mainly affect vulnerable groups; a form of marginalisation in not being considered knowledgeable or trustworthy because of who you are or are perceived to be.

## Methodological considerations

Focus groups are based on a collective understanding of participants’ views [27] and a crucial feature is to stimulate interaction between participants to create discussion. Fourteen researchers in ageing and health representing different disciplines such as the humanities, social sciences, medicine, and health care sciences participated in four focus groups. Several researchers claim that four, five groups are sufficient when working with specific target groups [25, 30, 46, 47], which was the case here. There were between three and five participants per focus group. Some authors argue that there should be six to twelve participants in each group, while others argue that the ideal number of participants is between four and eight [25, 30, 46, 47]. Thus, an issue to discuss when it comes to this study may be the limited number of participants in each group. We found that the discussions were dynamic, and the outcome of the discussions depended more on the involvement of the participants in each group than on the actual number of participants. The dynamic discussions may have been influenced by the fact that the participants represented different disciplines, were rather representative of the research area, and that the topic was of great importance to them. The focus group discussions allowed the participants to verbalise and share their experiences in an area they considered important and current. To obtain a broad representation of the target group and at the same time create an atmosphere that generates discussion, it is necessary to consider the heterogeneity and homogeneity within the groups [27]. What united our participants (homogeneity) was their common experience of being researchers, and the heterogeneity was based on the fact that they were from two different universities and represented different disciplines. Previous research has shown that being grouped with others with the same experiences, being able to discuss things with people who understand, and knowing that you are not the only one with a certain experience creates a sense of sharing [30, 48]. The participants in this study seemed to appreciate the opportunity to participate in the focus groups, which resulted in fruitful discussions where participants shared their opinions – both positive and negative. Negative opinions have been shown to be more easily expressed in the presence of other participants who have something in common [29, 49, 50]. This relates to the benefit of focus group methodology described as creating awareness [27].

## Conclusion

We want to highlight the researcher’s voice and clarify the role that researchers have in terms of the opportunities for older people to become part of the collective understanding of ageing and health and make their voices heard. As a researcher, it is important to strive for commitment from people that are as representative of the group as possible and to try, as far as possible, to understand the obstacles to fair representation that exist and take this into account in the presentation of results and conclusions. Lack of representativeness is a challenge for all research. The challenge for a researcher, as we see it, is to be a good navigator; to avoid the dominant social group imposing its worldview on the more vulnerable group, and at the same time avoid the risk of relativism that arises from treating the social group with less power only based on its minority position. A fruitful way forward [43] is an open dialogue to try to develop a way of understanding the world together. It means collaborating across boundaries to develop ways of thinking about important issues that we can all agree on. This dialogue should take place between open-minded people, who take the perspectives of others seriously, treat others as equal partners in the conversation, and are receptive to criticism of their own possible shortcomings. To collaborate with older people, researchers require certain competencies, such as the ability to build trust and communicate effectively. To address this, academia can provide supportive infrastructure, such as resources to build trust over time, that can help researchers develop these competencies. Funding agencies can also play a role in supporting collaborative research with older people. For instance, they can acknowledge that conducting research with older people who are not familiar with research is more time-consuming and may not fit into the time restrictions of funding. This can help ensure that researchers have the necessary resources to conduct high-quality research that involves older people. Based on the knowledge production of this study, we can plan how to proceed to the next step on how to conduct high-quality research to handle research challenges/biases regarding the heterogeneous group of older people, from healthy to very frail.

## Data Availability

The datasets generated and analysed during the current study are not publicly available due to the information provided to the involved persons when obtaining their informed consent, stating that all attempts would be made to maintain their confidentiality. De-identified data are available upon reasonable request to enable review and will be stored for 10 years from publication at the University of Gothenburg, Sweden. All data are covered by the Swedish Public Access to Information and Secrecy Act (offentlighets- och sekretesslagen) and a confidentiality assessment (sekretessprövning) will be performed at each individual request. Permission from the University of Gothenburg, the Institute of Neuroscience and Physiology, must be obtained before data can be accessed.
